# Unexpected early pulmonary thrombi in war injured patients

**DOI:** 10.1007/s00330-025-11925-2

**Published:** 2025-08-20

**Authors:** Itai Sasson, Vera Sorin, Tomer Ziv-Baran, Edith M. Marom, Evgenia Czerniawski, Sharon Z. Adam, Galit Aviram

**Affiliations:** 1 Departments of Radiology, Tel Aviv Sourasky Medical Center, Affiliated to the Faculty of Medical & Health Sciences, Tel Aviv University, Tel Aviv, Israel; 2 Department of Radiology, Sheba Medical Center, Affiliated to the Faculty of Medical & Health Sciences, Tel Aviv University, Tel Aviv, Israel; 3 School of Public Health, Faculty of Medical & Health Sciences, Tel Aviv University, Tel Aviv, Israel; 4 Department of Radiology, Barzilai Medical Center, Ben-Gurion University, Beer-Sheva, Israel

**Keywords:** Computerized tomography, Pulmonary embolism, Pulmonary thrombosis, Artificial intelligence

## Abstract

**Objectives:**

Pulmonary embolism is commonly associated with deep vein thrombosis and the components of Virchow’s triad: hypercoagulability, stasis, and endothelial injury. High-risk patients are traditionally those with prolonged immobility and hypercoagulability. Recent findings of pulmonary thrombosis (PT) in healthy combat soldiers, found on CT performed for initial trauma assessment, challenge this assumption. The aim of this study was to investigate the prevalence and characteristics of PT detected in acute traumatic war injuries, and evaluate the effectiveness of an artificial intelligence (AI) algorithm in these settings.

**Materials and methods:**

This retrospective study analyzed immediate post-trauma CT scans of war-injured patients aged 18–45, from two tertiary hospitals between October 7, 2023, and January 7, 2024. Thrombi were retrospectively detected using AI software and confirmed by two senior radiologists. Findings were compared to the original reports. Clinical and injury-related data were analyzed.

**Results:**

Of 190 patients (median age 24, IQR (21.0–30.0), 183 males), AI identified 10 confirmed PT patients (5.6%), six (60%) of whom were not originally diagnosed. The only statistically significant difference between PT and non-PT patients was increased complexity and severity of injuries (higher Injury Severity Score, median (IQR) 21.0 (20.0–21.0) vs 9.0 (4.0–14.5), *p* = 0.01, accordingly). Despite the presence of thrombi, significant right ventricular dilatation was absent in all patients.

**Conclusions:**

This report of early PT in war-injured patients provides a unique opportunity to characterize these findings. PT occurs more frequently than anticipated, without clinical suspicion, highlighting the need for improved radiologists’ awareness and the crucial role of AI systems as diagnostic support tools.

**Key Points:**

***Question*** *What is the prevalence, and what are the radiological characteristics of arterial clotting within the pulmonary arteries in young acute trauma patients?*

***Findings*** *A surprisingly high occurrence of PT with a high rate of missed diagnoses by radiologists. All cases did not presented right ventricular dysfunction*.

***Clinical relevance*** *PT is a distinct clinical entity separate from traditional venous thromboembolism, which raises the need for further investigation of the appropriate treatment paradigm*.

**Graphical Abstract:**

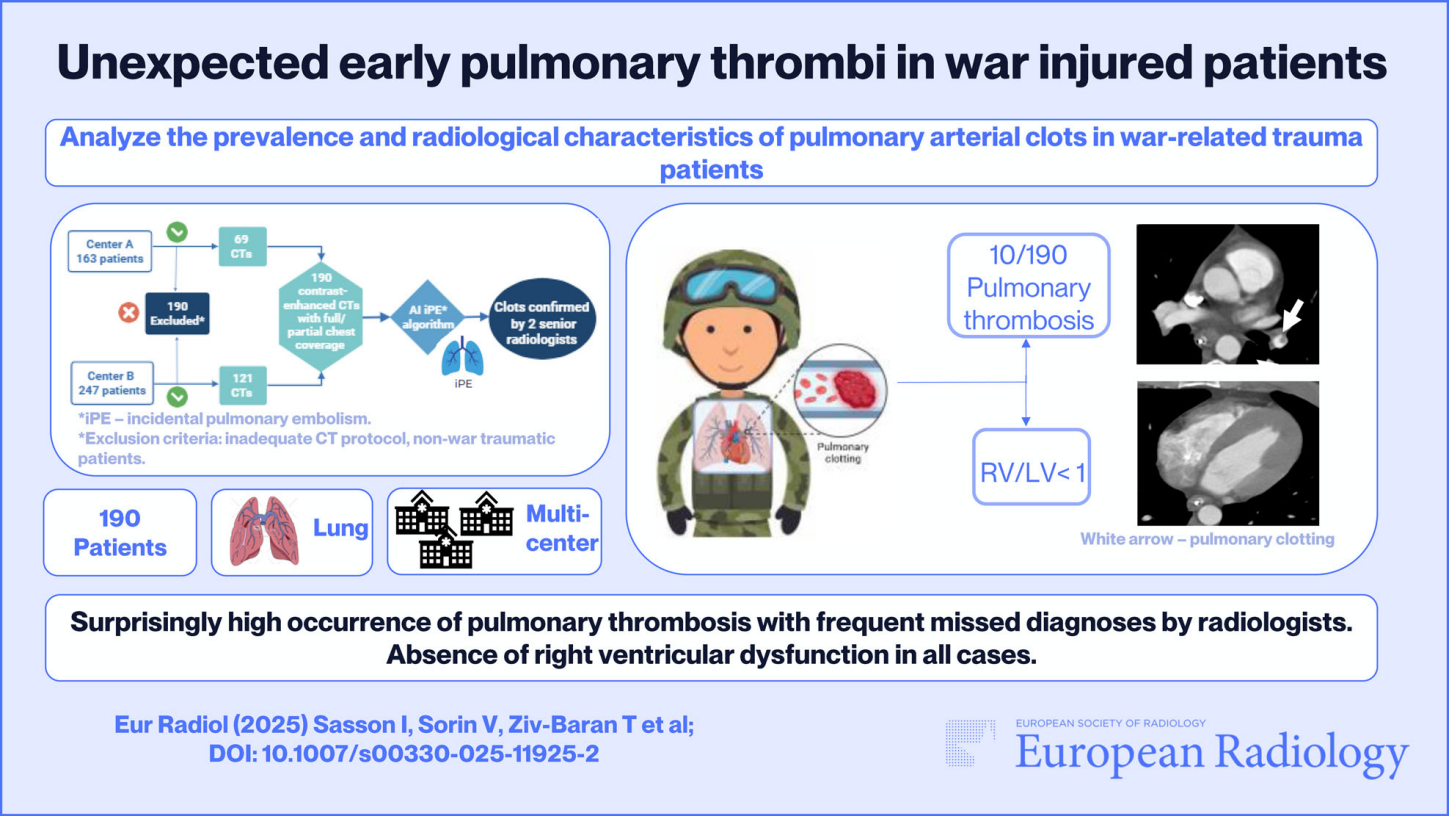

## Introduction

Traditionally, pulmonary embolism (PE) is thought of as the sequela of deep vein thrombosis (DVT), which is related to Virchow’s triad of hypercoagulability, stasis, and endothelial injury [1, 2]. Accordingly, risk factors for developing venous thromboembolism (VTE) are considered older age, prolonged immobility, heart failure, major trauma or surgery, lower-limb fractures or joint replacements, and spinal cord injury [3, 4]. Cancer is another well-recognized predisposing factor for VTE [5]. Moreover, the longstanding assumption that DVT and PE are manifestations of the same pathology, has driven prophylactic guidelines with the hypothesis that preventing DVT will lead to a reduction in PE [6]. It is also commonly assumed by radiologists worldwide that filling defects in the pulmonary arteries represent PE.

Recently, we encountered cases of pulmonary arterial filling defects seen on contrast-enhanced CT scans performed for evaluation of acute war injuries, at the time of admission, in young and healthy combat soldiers. Finding PE at such an early phase in healthy, active young individuals was unexpected. Although previous studies have reported cases of PE in trauma patients [7], we found no documented evidence in the literature describing such cases among acute war-injured patients. Because of the less likely mechanism of pulmonary embolism in our patient cohort, and the known hypercoagulable state encountered in early trauma [8, 9], we were suspicion of an in situ mechanism of thrombus formation within the pulmonary arteries, termed pulmonary thrombosis (PT), was the culprit in these patients, as opposed to the conventional embolic mechanism of pulmonary embolism. The aim of our study was to evaluate the prevalence of PT, characterize the radiological findings in terms of thrombi location and the response of the right ventricle (RV) to their presence, and assess symptoms, background injuries, and the outcome in terms of 7-day mortality.

This distinction between PT and PE as the cause for filling defects in the pulmonary arterial vasculature has a profound effect on the management of these very complicated multi-organ trauma patients. The diagnosis of PE in acute trauma patients can lead to the administration of anticoagulation or inferior vena cava (IVC) filter placement, as well as long-term VTE prevention treatment. Therefore, understanding the nature and pathophysiology of incidental fresh pulmonary thrombi is crucial.

Our second goal was to examine the contribution of an artificial intelligence (AI) algorithm in the diagnosis of pulmonary thrombi in acute trauma patients. It has been shown that a common radiology error is a history-related bias [10], in cases where a scan is performed for a specific clinical question, which is completely unrelated to a certain finding, as is the case in trauma patients and pulmonary arterial filling defects. In these cases, an AI algorithm that does not have this bias, can reduce radiological errors.

## Materials and methods

This study was conducted in accordance with the principles outlined in the Declaration of Helsinki and adhered to all applicable regulations regarding human rights. Institutional review board (IRB) approval was obtained in both of the involved medical centers (IRB approval numbers - TLV-0744-23 and SMC-D-0957-23). This is a retrospective study, and informed consent was waived.

### Study population

**Fig. 1:**
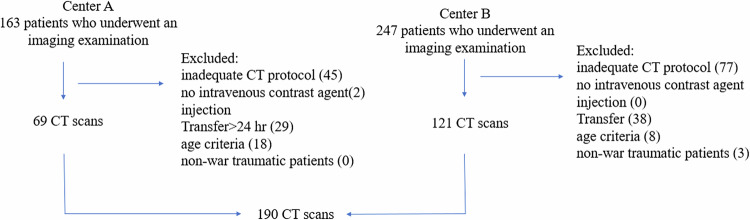
Flow chart illustrating the exam selection process across two centers. After applying exclusion criteria, 69 CT scans from Center A and 121 CT scans from Center B were analyzed

We collected cases from two tertiary medical centers, Tel Aviv Sourasky Medical Center and Sheba Medical Center, which served as initial trauma centers during the “October 7th attack” and its aftermath. We retrospectively evaluated all consecutive contrast-enhanced CT scans that were performed for the initial assessment of acutely injured war patients between October 7th, 2023, and January 7th, 2024. Our inclusion criteria were: male and female patients, ages 18–45 years, contrast-enhanced CT scans which included the entire chest, or part of the chest as can be seen in neck or abdominal studies, scans performed in the first 24 h following the injury, and trauma which was war-related only. Only imaging studies that met all inclusion criteria were included in the analysis (see Fig. 1), and all other scans were excluded.

Patients were transported to the hospital by helicopters, ambulances, or vehicles. Initial first aid and resuscitation were provided by the medical teams in the field and during transport. Based on the current treatment paradigm for PE, patients with a real-time diagnosis of PT received anticoagulation therapy or an IVC filter, according to their treating physicians’ decision.

### CT acquisition

Imaging was performed according to the mechanism of injury and the patient’s clinical presentation upon arrival. In most cases, patients underwent a routine trauma protocol that included the chest, abdomen, and sometimes the entire lower limbs, in the arterial phase. When a full trauma protocol was not medically indicated—such as in cases of isolated penetrating trauma to the head and neck or lower limbs—targeted imaging was performed based on clinical judgment. This typically included either the upper or lower lung fields, depending on the nature of the injury.

Patients were scanned by various multi-detector CT scanners available in each medical center’s emergency department. Reconstructed slice thickness was 1.0–3.0 mm according to the local institutional trauma protocol with injections of 80–100 mL of iodinated contrast material, at a concentration of 300–350 mg iodine per mL, and at rates of 3–4 mL/s. In both participating medical centers, bolus tracking was employed in the descending aorta as part of the standard trauma CT protocol. It is important to note that spectral CT was not available during the study period at either institution, and therefore was not used for the evaluation of the findings.

### CT assessment

The CT scans were immediately reviewed by a local senior radiologist at each site. AI algorithms for automatic detection of incidental pulmonary embolism (iPE) were not available for trauma protocols at that time. The AI algorithm was used retrospectively, as part of the study only, to confirm that no PT cases were missed. This commercially available deep learning cloud–based AI algorithm for PE detection and triage (Aidoc BriefCase, Aidoc Medical), has recently been shown to be effective for iPE detection [11]. When filling defects within the pulmonary arteries were reported by the primary reviewing radiologist or detected by the AI algorithm, the scan was re-reviewed by two senior radiologists at each site (E.M. and V.S., or G.A. and S.Z.A.), to confirm the real presence of thrombi. In all positive cases, a minimum threshold of luminal attenuation of 93 Hounsfield units (HU) was required in order to confirm a true positive diagnosis [12]. This value was measured by a region of interest (ROI) placed within the main pulmonary artery, or the RV, when there was only an abdominal CT.

If the case was deemed positive after the senior radiologists’ assessment, we measured the diameters of the RV and left ventricle (LV) on axial images by identifying the maximal distance between the ventricular endocardium and the interventricular septum, perpendicular to the long axis of the heart. The location of arterial thrombi and findings suggestive of RV dysfunction, including RV/LV ratio ≥ 1, septal flattening or bowing, substantial reflux of contrast into the IVC and hepatic veins, were measured and recorded according to the literature [13, 14]. In exams where only a partial scan of the chest was performed, the relevant parameters that could be extracted from the scan, such as the location of the thrombi and, if applicable, findings of RV dysfunction, were collected.

### iPE detection algorithm

In both centers, we utilized Aidoc’s FDA-cleared AI-based system for the detection of iPE. The algorithm continuously analyzes contrast-enhanced CT scans to identify suspected emboli, including in studies not dedicated to PE evaluation. When findings are detected by the algorithm, automated alerts are generated and integrated into the radiology workflow. The algorithm for detecting incidental findings differs from conventional PE detection algorithms in its ability to identify pulmonary emboli in CT scans not specifically performed with a pulmonary embolism protocol—such as trauma, abdominal, or neck scans. This is achieved through automated recognition of relevant protocols, including the administration of intravenous contrast and the inclusion of lung fields within the scan range. Previous studies have demonstrated high diagnostic accuracy of similar AI algorithms, with reported sensitivities and specificities of 91.6% and 99.7% [15, 16], respectively. In this study, the software was applied retrospectively after scan acquisition for research purposes only; the algorithm was not active during the initial clinical interpretation of the exams.

### Clinical information

In addition to gender and age, the electronic medical records were reviewed for the site and type of injury, including penetrating, blast, shrapnel, smoke inhalation, acoustic, and crush injuries. Patients could sustain more than one type of injury, therefore, the reported mechanisms are not mutually exclusive, and the same patient may be classified under multiple trauma mechanisms categories (e.g., blast and shrapnel). Prehospital treatments, such as ventilatory support and tourniquet usage, were recorded. The severity of injury was assessed by the injury severity scale (ISS) and by the presence of shock on admission, defined as SBP < 90 mmHg. Procedures performed in the emergency room, such as intubation, administration of packed cells (PC), chest tube insertion, high-flow nasal cannula usage, and emergency department thoracotomy (EDT), were also documented. Emergency surgery and admission to an intensive care unit (ICU) were recorded as well. Coagulopathy was defined by an international normalized ratio (INR) > 1.2; acute phase reaction was assessed by platelet count, and neutrophil-to-lymphocyte ratio. Respiratory parameters, including oxygen saturation, hyperventilation (> 20 breaths/min), and a sensation of dyspnea, were also recorded. Additionally, it was documented whether the imaging test included the entire chest or only part of it.

We reviewed clinical records for evidence of leg injuries or lower extremity DVT. Mortality at 7 days and admission to a rehabilitation institution were also recorded.

### Statistical analysis

Categorical data were summarized as frequency and percentage. Continuous variables distribution was evaluated using a histogram.

Since not all continuous data were normally distributed, they are presented as median and interquartile range. The Mann–Whitney test and Fisher’s exact test were applied to compare continuous and categorical variables, respectively, between patients with and without PT.

All statistical tests were two-sided, and *p* < 0.05 was considered statistically significant. Statistical analysis was performed using SPSS software (IBM SPSS Statistics for Windows, version 28, IBM Corp.).

## Results

The study population included 410 (163 in one center and 247 in the other) acutely war-injured patients from two tertiary trauma centers.

Following the exclusion of 220 individuals, (54%) due to inadequate CT protocol (no part of the chest field (*n* = 122), no intravenous contrast agent injection (*n* = 2), age criteria (*n* = 26), transfer from another hospital that occurred later than 24 h post-injury or following a surgical intervention (*n* = 67), and non-war trauma patients (*n* = 3), the final study cohort included 190 patients aged 18–45, who had a contrast-enhanced full or part of the chest scan within the first 24 h of their war injury. After executing the AI algorithm for PE identification on all 190 patients, the algorithm identified 10 patients with pulmonary thrombi (5.6%), all confirmed as filling defects within the pulmonary arteries by 2 senior radiologists (Fig. 1). The mean HU in the main pulmonary artery or the RV was 270.9, ranging from 124 to 369, thus, all measurements showed a luminal attenuation greater than 93 HU.

The baseline characteristics of the study population in all 190 patients, the non-PT group, and the PT group, are presented in Table 1.

**Table 1:** Patient demographic and clinical characteristics

	Overall (*n* = 190) *n*/median (%/IQR)	Non-PT (*n* = 180) *n*/median (%/IQR)	PT (*n* = 10) *n*/median (%/IQR)
Age at admission, median (IQR), y	24 (21.0–30.0)	24 (21.0–30.0)	21.5 (20.75–28.75)
Sex			
Males	183 (96.02)	173 (96.11)	10.0 (100)
Females	7 (3.68)	7 (3.89)	0.0 (0.0)
Center			
A	69 (36.32)	64 (35.56)	5 (50.0)
B	121 (63.68)	116 (64.44)	5 (50.0)
Air evacuation	155.0 (81.58)	146.0 (81.11)	10 (100.0)
Mechanism*			
Penetrating	63.0 (33.16)	62.0 (34.44)	1 (10.0)
Blast	74 (38.95)	68 (37.78)	6 (60)
Shrapnel	113 (59.47)	103 (57.22)	10 (100.0)
Other	24 (12.63)	24 (13.3)	0.0 (0.0)
Complexity			
1	114 (60)	111 (61.7)	3 (30)
2	68 (35.8)	61 (33.9)	7 (70)
3 ≤	8 (4.2)	8 (4.4)	0 (0)
Site			
Chest	45 (23.68)	42 (23.33)	3 (30)
Non chest	145 (76.3)	138 (76.6)	7 (70)
Lower limbs	78 (41.1)	71 (39.4)	7 (70)
Saturation, median (IQR)	100 (98–100)	99.5 (98–100)	100 (100–100)
Hyperventilation (breaths > 20)	33 (17.37)	31 (17.22)	2 (20)
Dyspnea	19 (10)	18 (10)	1 (10)
ISS score, median (IQR)	9.0 (4.25–19.25)	9.0 (4.0–14.5)	21.0 (20.0–21.0)
Shock on admission (SBP < 90 mmHg))	7 (3.68)	6 (3.3)	1 (10)
Laboratory tests			
INR > 1.5	7 (3.7)	7 (3.89)	0 (0)
PLT, median (IQR)	227 (184.5–272.5)	225.0 (187.0–272.0)	240.5 (148.5–318.5)
NLR, median (IQR)	4.53 (2.9–8.29)	4.47 (2.90–7.98)	8.0 (2.16–12.27)
Full CT chest	111 (58.42)	104 (57.78)	7 (70)
CT chest field	79 (41.15)	76 (42.22)	3 (30)
Treatment			
Tourniquet	54 (28.42)	47 (26.11)	7 (70.0)
Intubation	19 (10)	17 (9.44)	2 (20)
Packed cell transfusion	25 (13.16)	22 (12.22)	3 (30)
Chest tube	14 (7.37)	14 (7.78)	0.0 (0.0)
Admission to a rehabilitation institution	68 (35.79)	61 (33.89)	7 (70.0)

*PT* pulmonary thrombosis, *IQR* interquartile range, *ISS* injured severity scale

^*^ Note: injury mechanisms are not mutually exclusive; patients may appear in more than one category

There were 183 males and 7 females with a median age of 24 (21.0–30.0) years. The PT group included ten males and no females. Their median age was 21.5 (20.75–28.75) years. The age differences of the two groups were not significant (*p* = 0.545).

A higher injury severity score (ISS) was found in the PT group, 21.0 (20.0–21.0) vs 9.0 (4.0–14.5), respectively (*p* = 0.01), while there was no significant difference between the groups concerning the additional clinical and demographic parameters collected. Coexisting lower extremity injuries were documented and showed no significant difference between groups, although a non-statistically significant trend was observed, of a higher rate of lower extremity injuries in PT patients (70% compared with 39.4% in non-PT patients, *p* = 0.094; see Table 1). As all patients underwent imaging as part of the emergency department assessment shortly after injury, none underwent a dedicated venous ultrasound of the lower limbs for the evaluation of DVT. Consequently, sonographic data regarding DVT were not available, and analysis was limited to the presence of clinically documented limb injuries.

We did not observe differences in the types of trauma mechanisms among patients in the PT group compared to other injured individuals. However, recognizing that these patients are more complex, we decided to categorize the mechanisms of injury based on their complexity, specifically according to the number of injuries. Analysis by the number of injuries indicated a trend towards more complex injuries in the PT group (a trend that was not statistically significant, *p* = 0.077).

Additionally, various laboratory data collected, including platelet count, coagulation functions, and the ratio of neutrophils to lymphocytes (NLR ratio), did not demonstrate significant differences between the groups. Respiratory parameters such as oxygen saturation, respiratory rate, and dyspnea also did not show significant differences between the two groups.

Data was collected for all injured individuals regarding risk factors for developing VTE, including oncological history, pregnancy, and inflammatory bowel disease (IBD). As expected, in the group primarily consisting of combat soldiers, none of the participants had any of the risk factors mentioned.

Of the scans reviewed, 111 (58.5%) included full chest imaging with systemic arterial phase contrast as per the trauma protocol, while the remaining scans were limited to the lung apices or bases with contrast. In the group of PT patients, seven underwent a complete CT trauma protocol during the initial assessment, whereas the remaining three were identified through partial lung field scans.

In each center, AI identified filling defects in the pulmonary arteries of five trauma patients. In one center, 4 out of the 5 patients were identified by a senior radiologist during the initial official reading and reported. In the second medical center, none of the five cases were identified by the reporting senior radiologist. This means that 6 out of 10 cases were not recognized in real-time during the primary radiological review (60%).

Table 2 demonstrates various radiological parameters and treatment among the ten PT-positive patients’ group. Most of the filling defects were observed in a segmental or sub-segmental location. In all ten cases, there were no findings to suggest RV dysfunction.

**Table 2:** Radiologic characteristics of the pulmonary clots, RV strain assessment, and treatment among the patients with pulmonary thrombi

Measurements	Units	PT group			
		(*N* = 10)			
		Number	%	Median	IQR
Detection by a radiologist		4	40		
Site					
Main		0			
Lobar		3			
Segmental		8			
Sub-segmental		7			
Pulmonary trunk diameter				2.4	2.3–2.65
RV diameter	cm			3.8	3.05–4.25
LV diameter	cm			4.4	4.1–4.8
RV/LV RATIO				0.82	0.75–0.96
Reflux of contrast^*^		2			
Anticoagulation		4	40		
IV filter		1	10		

*RV* right ventricle, *LV* left ventricle, *IV* intravenous

^*^ Substantial reflux of contrast into the IVC and at least a trace in one of the hepatic veins

**Fig. 2:**
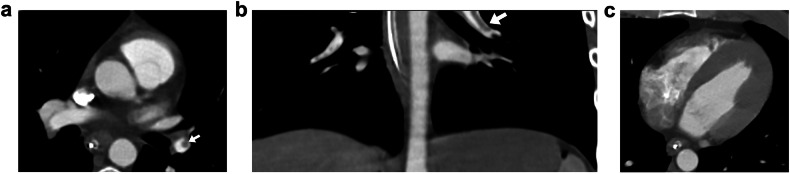
Panel graph outline. Example of a 31-year-old male soldier with a gunshot wound who was treated with an IVC filter following identification of pulmonary arterial clots on the CT. The initial assessment included an abdominal trauma CT protocol covering only the lower chest. **a**, **b** Axial and coronal views show a filling defect within the left lower lobar artery consistent with a pulmonary thrombus (PT) (arrows). **c** Axial view of the heart: the right to left ventricular ratio is normal

All patients identified by a radiologist during the initial interpretation received therapeutic-dose anticoagulant treatment for several months. None had bleeding complications. One patient out of the four underwent placement of an IVC filter (Fig. 2a, c). All identified patients underwent follow-up scans using a PE protocol after several months, and no thrombus was found. Among the 190 patients, no mortality was observed within 7 days of the injury.

## Discussion

This series is unique in describing the radiological characteristics of acute PT as incidental findings on initial trauma assessment CT scans. The presence of PT among acutely war-injured young active patients was unexpected since traditional risk factors for VTE were mostly absent.

Findings of central filling defects within the pulmonary arterial branches, which we always consider as “pulmonary embolism,” may represent pulmonary thrombi that are not embolic but rather in situ thrombi resulting from inflammation, endothelial injury, and the hypercoagulable state caused by the injury itself, thus termed “pulmonary thrombosis” as opposed to PE. These thrombi are de novo clots with distinct risk factors as very recently suggested by the Consortium of Leaders in the Study of Traumatic Thromboembolism (CLOTT) study group [7], who published a large prospective, multicenter cohort study of 7880 trauma patients from 17 US trauma centers providing highly supportive evidence that PT arises de novo without DVT, is often asymptomatic, and may be a manifestation of local inflammation, thus representing a distinct clinical entity associated with the nature of the injury, different from the traditional venous thromboembolic paradigm of DVT and PE.

Significant progress has been made in our understanding of in situ thrombosis within the pulmonary arteries, owing to recent breakthroughs in coagulation and thrombosis research. Recent studies have highlighted the crucial role of innate immunity and inflammation in coagulation, underscoring the convergence of immune reactions and thrombogenicity [17–19]. These insights offer a more comprehensive explanation for the occurrence of in situ PT in various proinflammatory conditions, such as acute trauma.

Similar to our findings, in recent years, there has been growing recognition that thromboembolic events in trauma patients may occur earlier than expected. A number of recent publications [20, 21] have demonstrated that VTE, including PE, can develop within the first few days following traumatic injury. This phenomenon has also been associated with acute post-traumatic inflammation as a fourth component to the genesis of thrombi beyond Virchow’s triad.

Studies have examined trauma-induced coagulopathy (TIC) mechanisms [8, 9], and the patient response timeline is divided into an early phase (within 6 h) which is dominated by hypocoagulability, a delayed phase (beyond 24 h) which is dominated by hypercoagulability, and an intermediate phase between 6 and 24 h which is a transition phase between the two tendencies. Our findings of thrombosis in the first 24 h possibly support a stronger role of the hypercoagulability type of TIC in the early and transition phases than previously thought, potentially related to different types of trauma mechanisms than those described in the literature for non-war-related injuries. This, of course, warrants further investigation with a larger cohort, for a better understanding of coagulopathies in different types of trauma.

Recognizing PT as a distinct clinical entity with its own pathogenicity, separate from PE, raises doubts regarding the treatment approach of these conditions as traditional PE.

Importantly, these recommendations hold particular relevance for patients at an increased risk of complications related to anticoagulation. In our study, although the incidence of PT events was high (5.6%), none of the patients demonstrated radiological evidence of RV dysfunction. This absence may be attributed to the predominantly peripheral location of the thrombi. Most clots were found at the segmental or subsegmental level, with no involvement of the main or lobar pulmonary arteries. This distribution is consistent with existing radiological evidence (13), which indicates that RV strain is typically associated with central or high clot burden pulmonary emboli, whereas peripheral thrombi rarely lead to hemodynamic compromise. Radiological signs of RV dysfunction are generally absent in such cases. This suggests that while thrombi were present, they did not cause hemodynamic consequences. This finding raises important questions regarding the clinical significance of iPT detected in trauma patients and whether these thrombi warrant immediate anticoagulation or invasive interventions such as IVC filter insertion. Given that the patients did not exhibit traditional signs of RV strain or significant hemodynamic compromise, nor evidence of DVT, the decision to initiate treatment for these incidental findings requires careful consideration.

Nevertheless, this finding underscores the need for further investigation into the management strategies for such events. The CLOTT study suggests that these cases should be addressed by focusing on two pivotal parameters: Firstly, clinical manifestations and symptoms. Secondly, the location of the thrombus. Guided by these criteria, they recommend initiating treatment when the thrombus is at the segmental level or more proximal, or when there is a clinically evident presentation. If the thrombus is subsegmental and asymptomatic, a watchful management approach is encouraged [7].

Another important issue that our study raises is that radiologists’ awareness of PT in trauma cases was poor. In 60% of cases, these events were missed by the radiologist during real-time patient evaluation, while the AI algorithm successfully identified them. These misses likely reflect perceptual errors which are related to thinking bias due to satisfaction of search, or history-relates bias (an assumed low pre-test probability for pulmonary embolism in acute trauma patients) which leads to decreased vigilance to other abnormalities after detecting or ruling-out trauma-related injuries, resulting in the termination of reading and the omission of other vital but unexpected findings [10]. Therefore, it is essential to increase the radiologists’ awareness of PT, even in patients without any known risk factors for VTE, especially in trauma cases with severe and complex mechanisms. Early detection of such events will allow for the development of more appropriate and effective treatment strategies for these patients.

A question can be raised regarding the importance of recognizing small asymptomatic PT, and the need for radiologists' awareness of this entity. Firstly, awareness is crucial in order to prevent unnecessary recommendations for treatment. Secondly, since thrombosis can deteriorate into a clinically significant state, it is crucial that the treating physicians are aware of all risk factors in critically ill trauma patients. Furthermore, accurate reporting of these findings can lead to more extensive research into this subject, thus allowing for a better understanding of the underlying mechanisms, furthering medical knowledge.

The limitations of our study primarily stem from its retrospective nature. Although no significant differences were found in demographic and clinical data between the comparison groups, we cannot rule out other selective biases. This challenge is particularly evident when working with a small cohort, as in our study, even though data were collected from two tertiary medical centers. Due to the limited cohort size, only a small number of positive cases were found. It is important to note that although the AI algorithm has a sensitivity of approximately 90% in detecting pulmonary filling defects, the actual incidence of thrombosis may be even higher, as some cases might have gone undetected. Moreover, while 58.4% of patients underwent full chest CT scans, a significant proportion (41.6%) had only partial chest imaging—typically limited to the apices or bases of the lungs as part of neck or abdominal CT studies. In these cases, key segments of the pulmonary vasculature may not have been fully visualized, potentially resulting in missed pulmonary thrombi by both radiologists and the AI algorithm. Furthermore, beyond these partial evaluations, some patients—who sustained no significant traumatic mechanism and remained asymptomatic—may not have undergone any CT imaging at all, based on the clinical judgment of the attending physician. Thus, it is possible that additional cases may have existed, unbeknownst to us. It is also important to note that during this national mass casualty event, the medical record documentation—particularly from data collected in the early phases of the study—was incomplete, especially regarding the treatment provided in the field. Most participants underwent CT scans using trauma protocols, which included an arterial phase; however, no patient underwent CT pulmonary angiography (CTPA) as part of the initial evaluation.

In conclusion, this study provides novel insights into the prevalence and characteristics of PT in young, healthy trauma patients, challenging traditional assumptions about the risk factors for VTE.

In this study, we were able to demonstrate a high incidence of PT, reaching 5.6% among war-related trauma patients, and to provide an initial characterization of the radiological features associated with these events. The use of AI algorithms for PT detection offers an important advancement to improving diagnostic sensitivity, and helps draw the radiologist’s attention to positive findings, even outside the immediate clinical context being considered. AI may also offer insights into processes related to unexpected phenomena when noticed, as demonstrated in this study. In our opinion, AI is mainly used as a supportive tool for decision-making and enhancing search strategies, especially in reducing the satisfaction of search bias. To be truly effective, it should be used alongside comprehensive radiological education that emphasizes pathophysiological understanding and active diagnostic thinking.

## References

[1] Essien EO, Rali P, Mathai SC (2019) Pulmonary embolism. Med Clin N Am 103:549–564 30955521 10.1016/j.mcna.2018.12.013

[2] Bagot CN, Arya R (2008) Virchow and his triad: a question of attribution. Br J Haematol 143:180–190 18783400 10.1111/j.1365-2141.2008.07323.x

[3] Rogers MA, Levine DA, Blumberg N, Flanders SA, Chopra V, Langa KM (2012) Triggers of hospitalization for venous thromboembolism. Circulation 125:2092–2099 22474264 10.1161/CIRCULATIONAHA.111.084467 PMC3342773

[4] Anderson FA, Spencer FA (2003) Risk factors for venous thromboembolism. Circulation 107:I9–I16 12814980 10.1161/01.CIR.0000078469.07362.E6

[5] Chew HK, Wun T, Harvey D, Zhou H, White RH (2006) Incidence of venous thromboembolism and its effect on survival among patients with common cancers. Arch Intern Med 166:458–464 16505267 10.1001/archinte.166.4.458

[6] Konstantinides SV, Meyer G, Becattini C et al (2020) 2019 ESC guidelines for the diagnosis and management of acute pulmonary embolism developed in collaboration with the European Respiratory Society (ERS). Eur Heart J 41:543–603 31504429 10.1093/eurheartj/ehz405

[7] Knudson MM, Moore EE, Kornblith LZ et al (2022) Challenging traditional paradigms in posttraumatic pulmonary thromboembolism. JAMA Surg 157:e216356 34910098 10.1001/jamasurg.2021.6356 PMC8674801

[8] Martini WZ (2016) Coagulation complications following trauma. Mil Med Res 3:35 10.1186/s40779-016-0105-2 PMC5120509 27895932

[9] Moore EE, Moore HB, Kornblith LZ et al (2021) Trauma-induced coagulopathy. Nat Rev Dis Prim 7:30 33927200 10.1038/s41572-021-00264-3 PMC9107773

[10] Zhang L, Wen X, Li JW, Jiang X, Yang XF, Li M (2023) Diagnostic error and bias in the department of radiology: a pictorial essay. Insights Imaging 14:163 37782396 10.1186/s13244-023-01521-7 PMC10545608

[11] Wiklund P, Medson K, Elf J (2023) Incidental pulmonary embolism in patients with cancer: prevalence, underdiagnosis, and evaluation of an AI algorithm for automatic detection of pulmonary embolism. Eur Radiol 33:1185–1193 36002759 10.1007/s00330-022-09071-0 PMC9889421

[12] Wittram C (2007) How I do it: CT pulmonary angiography. AJR Am J Roentgenol 188:1255–1261 17449768 10.2214/AJR.06.1104

[13] Araoz PA, Gotway MB, Trowbridge RL et al (2003) Helical CT pulmonary angiography predictors of in-hospital morbidity and mortality in patients with acute pulmonary embolism. J Thorac Imaging 18:207–216 14561905 10.1097/00005382-200310000-00001

[14] Aviram G, Rogowski O, Gotler Y et al (2008) Real-time risk stratification of patients with acute pulmonary embolism by grading the reflux of contrast into the inferior vena cava on computerized tomographic pulmonary angiography. J Thromb Haemost 6:1488–1493 18638012 10.1111/j.1538-7836.2008.03079.x

[15] Mohanarajan M, Salunke PP, Arif A et al (2025) Advancements in machine learning and artificial intelligence in the radiological detection of pulmonary embolism. Cureus 17:e78217 40026993 10.7759/cureus.78217 PMC11872007

[16] Kligerman SJ, Lahiji K, Galvin JR, Stokum C, White CS (2014) Missed pulmonary emboli on CT angiography: assessment with pulmonary embolism–computer-aided detection. AJR Am J Roentgenol 202:65–73 24370130 10.2214/AJR.13.11049

[17] Yong J, Toh CH (2023) Rethinking coagulation: from enzymatic cascade and cell-based reactions to a convergent model involving innate immune activation. Blood 142:2133–2144 37890148 10.1182/blood.2023021166

[18] Bahloul M, Dlela M, Bouchaala K et al (2020) Post-traumatic pulmonary embolism: incidence, physiopathology, risk factors of early occurrence, and impact outcome. Am J Cardiovasc Dis 10:432–443 33224594 PMC7675152

[19] van Dam LF, Kroft LJM, van der Wal LI et al (2020) Clinical and computed tomography characteristics of COVID-19 associated acute pulmonary embolism: A different phenotype of thrombotic disease? Thromb Res 193:86–89 32531548 10.1016/j.thromres.2020.06.010 PMC7274953

[20] Lineberry C, Alexis D, Mukhi A et al (2023) Venous thromboembolic disease in admitted blunt trauma patients: What matters? Thrombosis J 21:111 10.1186/s12959-023-00555-7 PMC10604411 37891537

[21] Arhoun el Haddad I, El Mouhib A, Hattab O et al (2022) Early bilateral pulmonary embolism in a polytrauma patient: about a case report. Ann Med Surg 78:103868 10.1016/j.amsu.2022.103868 PMC9207082 35734707

